# A T-CNN time series classification method based on Gram matrix

**DOI:** 10.1038/s41598-022-19758-5

**Published:** 2022-09-21

**Authors:** Junlu Wang, Su Li, Wanting Ji, Tian Jiang, Baoyan Song

**Affiliations:** School of Information, Liaoning University, Shenyang, 110036 China

**Keywords:** Electrical and electronic engineering, Mechanical engineering, Engineering

## Abstract

Time series classification is a basic task in the field of streaming data event analysis and data mining. The existing time series classification methods have the problems of low classification accuracy and low efficiency. To solve these problems, this paper proposes a T-CNN time series classification method based on a Gram matrix. Specifically, we perform wavelet threshold denoising on time series to filter normal curve noise, and propose a lossless transformation method based on the Gram matrix, which converts the time series to the time domain image and retains all the information of events. Then, we propose an improved CNN time series classification method, which introduces the Toeplitz convolution kernel matrix into convolution layer calculation. Finally, we introduce a Triplet network to calculate the similarity between similar events and different classes of events, and optimize the squared loss function of CNN. The proposed T-CNN model can accelerate the convergence rate of gradient descent and improve classification accuracy. Experimental results show that, compared with the existing methods, our T-CNN time series classification method has great advantages in efficiency and accuracy.

## Introduction

A time series is the result of a set of sequence data observed for a potential process at a given sampling rate in an equal period^1^. In recent years, with the development of computer technology, time series have become more and more widely applied, such as disaster monitoring, safety analysis, climate change, stock funds and other fields. How to classify time series^2–4^ has been a difficult point in the field of data mining. For example, mine disaster monitoring and early warning systems use microseismic sensors deployed around mines to store real-time data. These mine disaster events usually last from a few seconds to more than 10 s. Classifying these events helps to summarize the feature of various types of disasters^5^, which is of great significance for data analysis and disaster prevention.

The existing time series classification methods mostly use symbolic aggregation approximation (SAX)^6^ and convolution neural network (CNN)^7, 8^, but ignore the time attribute and the classification accuracy is not high. Therefore, to solve the above problems, this paper proposes a T-CNN time series classification method based on the Gram matrix^9^. CNN network replaces large-size convolution kernels by cascading multiple small-size convolution kernels, so that neurons of multiple small convolution kernels and one large convolution kernel have the same perception, which not only effectively reduces the number of network parameters, but also increases the nonlinear transformation and network depth of the network. It can extract more complex and abstract advanced features and improve the accuracy of model classification. The proposed method preserves the time attribute, and improves the square loss function^10^ of the CNN model in the full connection layer to enhance classification accuracy and efficiency. The main contributions of this paper are as follows:

- Aiming at the problem of loss of time attribute after transformation of time series matrix, a transformation method based on the Gram matrix is proposed, which converts time series into time-domain images without loss, and uses the wavelet threshold denoising method to filter out normal background noise^11, 12^.
- In order to increase the network convergence rate, a Toeplitz convolution kernel matrix^13^ is introduced into the CNN convolution layer, and the product of the Toeplitz matrix is used to replace the traditional convolution operation.
- A T-CNN model is proposed, which introduces a triplet network^14^ in the fully connected layer to calculate the difference function between the same class and different classes, and optimizes the square loss function of the CNN model, to improve the classification accuracy.

## Related work

At present, researchers have studied the time series classification and obtained some research achievements.

Zhong et al.^15^ proposed SAX, which divided the series into segments and converted each segment into a corresponding letter for classification. This method used the idea of aggregation to effectively reduce the dimension and increase the classification efficiency. However, this method lost a large number of data points in the time series, and the classification accuracy of this method was not high. Xing et al.^16^ proposed a trend turning point (TTP) method, which extracted the trend feature of the time series itself and required statistical analysis of the extreme points and inflection points of each time series. This method had a good effect on the data sets with specific rules, but was not good for the data sets with large trend fluctuations.

Marion et al.^17^ proposed a time domain distance (TDD) method, which reflected the similarity by calculating the Euclidean distance between different sequences. The closer the distance, the higher the similarity. The classification speed of this method was fast, but the classification accuracy was low. Marco et al.^18^ proposed an interval classification method, which divided time series into equal-length intervals, calculated the mean value and standard deviation of each interval, and used support vector machines for classification. This method regarded time series as a set of discrete data points and lost the time attribute.

Francisco et al.^19^ proposed a Shapelet method to find the Shapelet from the time series of known class labels to reflect the corresponding classification features. This method has high classification accuracy, but the time complexity of Shapelet extraction is high, and the classification efficiency is low. Chen et al.^20^ proposed a classification method based on convolution neural networks, which used CNNs to effectively classify time series. However, the training process of this method was complicated, and did not consider the time attribute.

To solve the above problems, this paper proposes a T-CNN time series classification method based on the Gram matrix, which converts time series into time-domain images without loss and retains time attributes. The proposed T-CNN model based on convolution neural networks inputs the time-domain images into the model, improving the classification accuracy.

## Gram matrix conversion of time series

Time series events are not one or a few abnormal perception data points, but a series of discrete data points that meet the threshold range in the time domain, and the same class events have similar features.

Definition 1 Time series events: A collection of continuous abnormal data initiated by the first outlier in the time series that exceeds the threshold range and lasts for a period of time. It can be represented as:

1 $$ E = \{ \left( {e_{1} ,e_{2} ,e_{3} ,...,e_{n} } \right)\left| {\forall i \in \left( {1,2,3,...,n} \right),} \right|e_{i} | > \phi \} $$

where $e_{i}$ represents an outlier in an event, and $\phi$ denotes the given threshold range.

According to Definition 1, each time series event has time attributes. Aiming at the lack of time attribute in the existing time series classification methods, we introduce the Gram matrix conversion method to realize the lossless conversion of time series.

### Normal noise filtering

After Gram matrix conversion, the time-domain image histogram presents normal distribution. The existence of normal noise directly affects the conversion of images in the time domain. Therefore, before the Gram matrix is used to convert the time series, the data is preprocessed first, and then the wavelet threshold denoising method is used to filter out the normal background noise carried by the data.

The steps of the wavelet threshold denoising method are as follows:

1. Wavelet decomposition of signals. A wavelet is selected to determine the *S*-level wavelet decomposition, and then the *S*-level wavelet decomposition calculation is carried out for the signals.
2. Threshold quantization of high-frequency coefficients in wavelet decomposition. From layer 1 to layer *S*, a threshold is selected for the high-frequency coefficients of each layer for threshold quantization. The threshold function used in this paper is calculated by:
  2 $$ \lambda = \sigma \sqrt {2\ln N} $$
  where $\sigma = M/0.6745$ denotes the median value of the absolute value of the first-level wavelet decomposition coefficient. Because the gaussian distribution function P(|x|< 0.6745σ) is approximately equal to 0.5, therefore, 0.6745 is the adjustment coefficient of the Gaussian noise standard deviation, and is a fixed value. *N* denotes the length of the signal.
3. Wavelet reconstruction of signals. According to the low-frequency coefficients of the *S* layer and the quantized high-frequency coefficients of the first layer to the *S*-th layer of the wavelet decomposition, the signal is reconstructed by wavelet.

**Figure 1:**
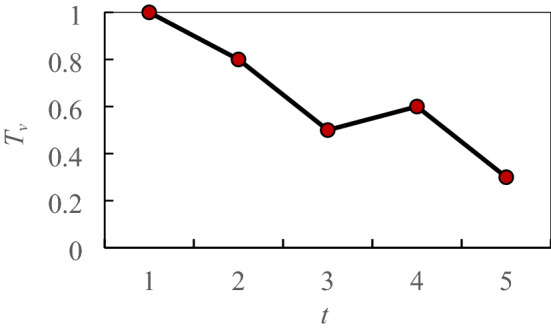
Time series.

Similar time series events have the same features, but the locations and intensities of the events are different, and the perceived event data are not at the same scale. Therefore, the normalization equation is used to keep the weight of the data feature dimension on the objective function consistent. The data normalization equation is as follows:

3 $$ x^{\prime} = \frac{{x - x_{min} }}{{x_{max} - x_{min} }} $$

where *x* denotes the data to be normalized, *x*´ denotes the normalized result, *x*_min_ represents the minimum value in the time series data, and *x*_max_ represents the maximum value in the time series data. Through data normalization conversion, all data can be normalized to [0,1]. For example, Fig. 1 shows a normalized time series *T*´ = {*t*_*1*_´, *t*_*2*_´, *t*_*3*_´,…, *t*_*n*_´} calculated by Eq. (3), the abscissa is time *t* and the ordinate is threshold *Tv*.

### Time domain image conversion based on Gram matrix

The time attribute is an important attribute to determine the classification. Due to the different time attributes, different regular distributions are presented in chronological order. Therefore, this paper proposes a time-domain image conversion based on the Gram matrix. This method can retain the time attributes and convert a time series into an *N* × *N* two-dimensional matrix without loss.

The Gram matrix *G* is a matrix composed of the inner product of each pair of vectors in the form of Eq. (4):

4 $$ G = \left( {\begin{array}{*{20}c} {\left\langle {a_{1} ,a_{1} } \right\rangle } & {\left\langle {a_{1} ,a_{2} } \right\rangle } & {...} & {\left\langle {a_{1} ,a_{n} } \right\rangle } \\ {\left\langle {a_{2} ,a_{1} } \right\rangle } & {\left\langle {a_{2} ,a_{2} } \right\rangle } & {...} & {\left\langle {a_{2} ,a_{n} } \right\rangle } \\ {\begin{array}{*{20}c} . \\ . \\ . \\ \end{array} } & {\begin{array}{*{20}c} . \\ . \\ . \\ \end{array} } & {\begin{array}{*{20}c} . \\ . \\ . \\ \end{array} } & {\begin{array}{*{20}c} . \\ . \\ . \\ \end{array} } \\ {\left\langle {a_{n} ,a_{1} } \right\rangle } & {\left\langle {a_{n} ,a_{2} } \right\rangle } & {...} & {\left\langle {a_{n} ,a_{n} } \right\rangle } \\ \end{array} } \right) $$

where < *a*_*i*_, *a*_*j*_ > denotes the inner product of two vectors.

The matrix G is a positive definite matrix. The proof of G is as follows:

Let $$Z = (x_{1} ,x_{2} ,...x_{n} )^{T} \ne 0$$, then

$$ Z^{\prime}GZ = \left( {x_{1} ,x_{2} ...x_{n} } \right)\left( {\begin{array}{*{20}c} {\left\langle {a_{1} ,a_{1} } \right\rangle } & {\left\langle {a_{1} ,a_{2} } \right\rangle } & {...} & {\left\langle {a_{1} ,a_{n} } \right\rangle } \\ {\left\langle {a_{2} ,a_{1} } \right\rangle } & {\left\langle {a_{2} ,a_{2} } \right\rangle } & {...} & {\left\langle {a_{2} ,a_{n} } \right\rangle } \\ {\begin{array}{*{20}c} . \\ . \\ . \\ \end{array} } & {\begin{array}{*{20}c} . \\ . \\ . \\ \end{array} } & {\begin{array}{*{20}c} . \\ . \\ . \\ \end{array} } & {\begin{array}{*{20}c} . \\ . \\ . \\ \end{array} } \\ {\left\langle {a_{n} ,a_{1} } \right\rangle } & {\left\langle {a_{n} ,a_{2} } \right\rangle } & {...} & {\left\langle {a_{n} ,a_{n} } \right\rangle } \\ \end{array} } \right)\left( {\begin{array}{*{20}c} {x_{1} } \\ {x_{2} } \\ \vdots \\ {x_{n} } \\ \end{array} } \right) $$

Thus,

$$ Z^{\prime}GZ = \left( {\mathop \sum \limits_{i = 1}^{n} a_{i} x_{i} ,\mathop \sum \limits_{i = 1}^{n} a_{i} x_{i} } \right) \ge 0 $$

where *Z*´*GZ* represents a positive definite quadratic form, and *G* denotes a positive definite matrix. A positive definite matrix can retain the feature of the matrix by calculating the eigenvalues. After converting the time series by the Gram matrix, the time attributes can be retained. The result of conversion to Gram matrix form is shown in Eq. (5):

5 $$ G_{t} = \left( {\begin{array}{*{20}c} {\left\langle {t_{1} ,t_{1} } \right\rangle } & {\left\langle {t_{1} ,t_{2} } \right\rangle } & {...} & {\left\langle {t_{1} ,t_{n} } \right\rangle } \\ {\left\langle {t_{2} ,t_{1} } \right\rangle } & {\left\langle {t_{2} ,t_{2} } \right\rangle } & {...} & {\left\langle {t_{2} ,t_{n} } \right\rangle } \\ {\begin{array}{*{20}c} . \\ . \\ . \\ \end{array} } & {\begin{array}{*{20}c} . \\ . \\ . \\ \end{array} } & {\begin{array}{*{20}c} . \\ . \\ . \\ \end{array} } & {\begin{array}{*{20}c} . \\ . \\ . \\ \end{array} } \\ {\left\langle {t_{n} ,t_{1} } \right\rangle } & {\left\langle {t_{n} ,t_{2} } \right\rangle } & {...} & {\left\langle {t_{n} ,t_{n} } \right\rangle } \\ \end{array} } \right) $$

where *G*_*t*_ denotes the time series after Gram matrix conversion, < *t*_*i*_, *t*_*j*_ > is the inner product pair of the time series, and *n* is the length of the time series. An inner product represents the correlation between two points, *G*_*t*_ a real symmetric matrix. From left to right of the first row and from top to bottom of the first column in the *G*_*t*_, the correlation between the first point and the subsequent points of the time series increases with time. Similarly, the second row and the second column are the correlation between the second point and the subsequent points. Therefore, the *G*_*t*_ matrix from the upper left to the low right represents the order of correlation arrangement between two points with time increasing. The time attribute of the time series is retained in the Gram matrix.

Since the time series *T* is one-dimensional, and in the rectangular coordinate system, the inner product pair calculation requires two-dimensional information of the abscissa and the ordinate. Therefore, to better preserve the time attribute of the time series, we use the polar coordinate system to calculate the inner product pairs of the time series.

The time series *T* can be encoded into polar coordinates by Eqs. (6 and 7):

6 $$ \theta_{i} = \arccos (t_{i}^{^{\prime}} ) $$

7 $$ r_{i} = \frac{i}{n} $$

where $t_{i}^{^{\prime}}$ denotes the normalized time series, *i* is the time stamp in the time series, *n* is the length of the time series, $\theta_{i}$ is the angle of a point in the time series in the polar coordinate system, and $r_{i}$ is the radius of a point in the time series in the polar coordinate system. Thus, each point in the time series can be represented by Eqs. (6 and 7).

**Figure 2:**
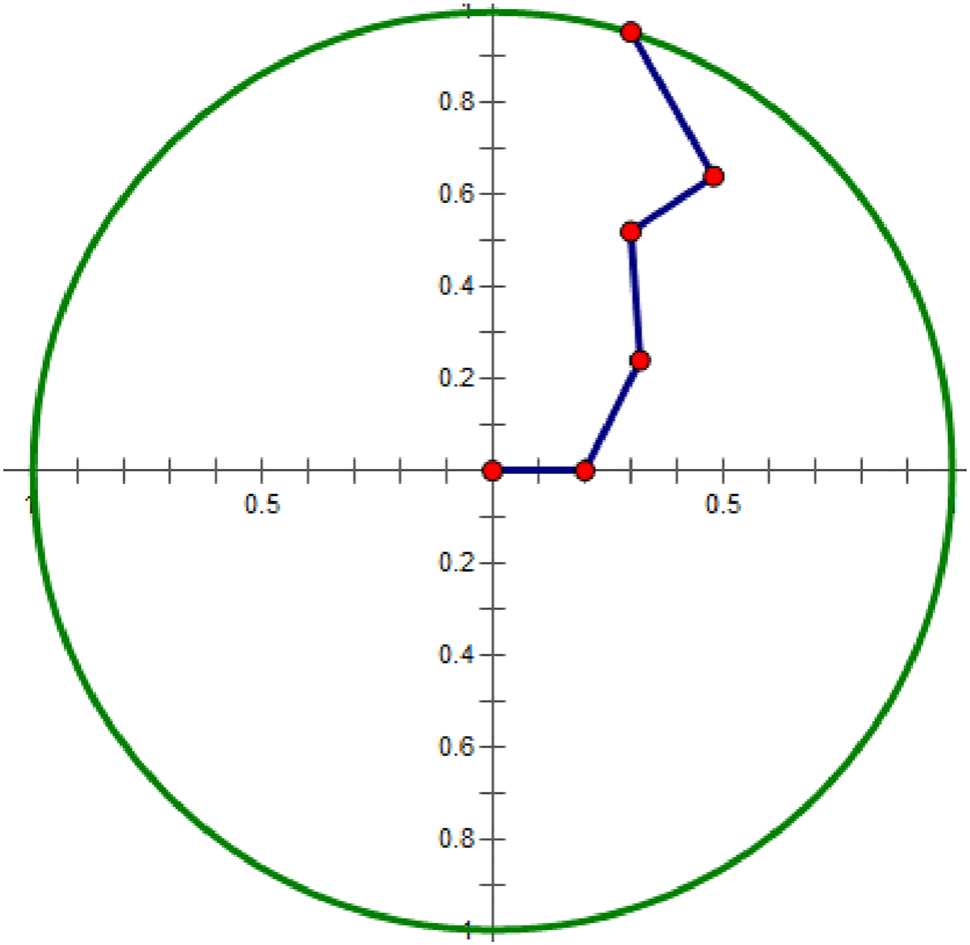
Polar coordinates of time series.

Figure 2 shows the polar coordinate coding of *T*´ in Fig. 1. The encoding process for 0.5 is:

According to Eq. (6), *arccos*(0.5) = 60º; *i* = 3; *N* = 5; according to Eq. (7), the radius is 0.6. Thus, (0.6, 60º) is the coding result.

As time goes on, the radius of the point becomes larger and larger, and gradually moves away from the center of the circle. Polar coding can completely preserve the time attribute by increasing the radius, and the numerical change can be represented by the angle change. Therefore, as shown in Eq. (8), a new Gram matrix can be obtained by using the polar coordinate angle relationship between two points in the time series:

8 $$ G_{t} = \left( {\begin{array}{*{20}c} {\cos (\theta_{1} + \theta_{1} )} & {\cos (\theta_{1} + \theta_{2} )} & {...} & {\cos (\theta_{1} + \theta_{n} )} \\ {\cos (\theta_{2} + \theta_{1} )} & {\cos (\theta_{2} + \theta_{2} )} & {...} & {\cos (\theta_{2} + \theta_{n} )} \\ {\begin{array}{*{20}c} . \\ . \\ . \\ \end{array} } & {\begin{array}{*{20}c} . \\ . \\ . \\ \end{array} } & {\begin{array}{*{20}c} . \\ . \\ . \\ \end{array} } & {\begin{array}{*{20}c} . \\ . \\ . \\ \end{array} } \\ {\cos (\theta_{n} + \theta_{1} )} & {\cos (\theta_{n} + \theta_{2} )} & {...} & {\cos (\theta_{n} + \theta_{n} )} \\ \end{array} } \right) $$

where $\cos (\theta_{i} + \theta_{j} ) = \cos \theta_{i} \cos \theta_{j} - \sin \theta_{i} \sin \theta_{j} = t_{i} \times t_{j} - \sqrt {1 - t_{i}^{2} } \times \sqrt {1 - t_{j}^{2} }$, and $\theta_{i}$ denotes the angle obtained by Eq. (6). When *i* = *j* is formed the diagonals $\cos (\theta_{i} + \theta_{i} ) = 2t_{i}^{2} - 1$ of the *G*_*t*_ matrix, and the diagonal of the *G*_*t*_ matrix is arranged in order.

As the position of the Gram matrix moves from the upper left corner to the lower right corner, the time series values are arranged into the matrix sequentially. In other words, we preserve the time attribute of the time series, and encode the time dimension into the geometric structure of the matrix. Each value of the matrix is equivalent to the pixel of the image, and each time series is converted into a time-domain image by the Gram matrix.

## Time domain image T-CNN classification

As mentioned above, time series are converted to Gram time-domain images, and the Gram time-domain images are used as the input matrix of convolutional neural networks for classification. In order to solve the problems of complex computation and slow training speed of convolutional neural networks, we propose a method based on the Toeplitz matrix product to replace the convolution operation of the convolution layer, and introduce the idea of triplet network into the loss function to improve the efficiency and accuracy of classification.

### Convolution based on Toeplitz matrix multiplication

**Figure 3:**
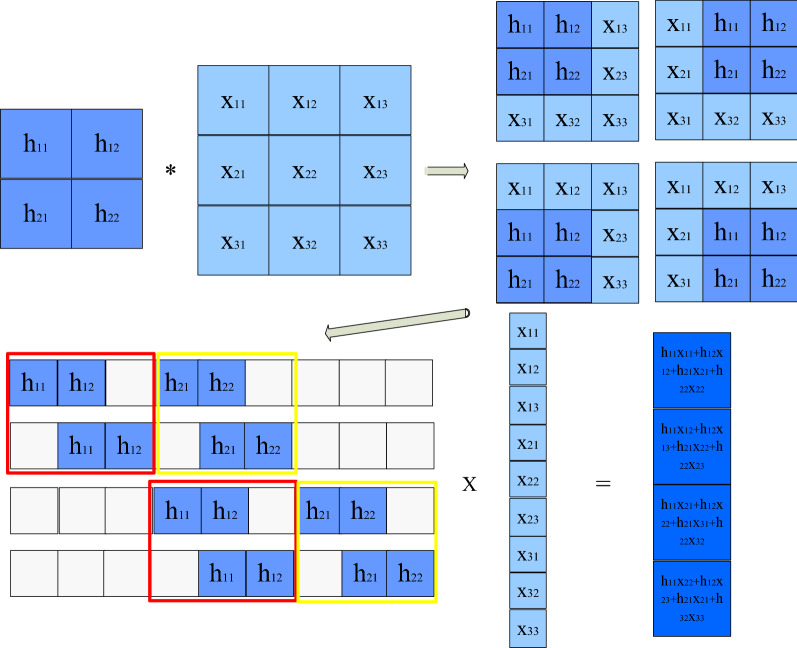
Convolutional computation.

The convolution operation based on the Toeplitz matrix product is shown in Fig. 3. In Fig. 3, the dark blue square represents the convolution kernel, and the light blue square represents the matrix being convoluted. The convolution kernel is 2 × 2, the unconvoluted matrix is 3 × 3, and the step size is 1. The traditional convolution is shown in the upper part of Fig. 3. The convolution kernel moves successively on the matrix to be convoluted according to the step size of 1, and it requires four traversals of the complete matrix to be convoluted. After each traversal, the convolution kernel and the matrix part with its repeated sum are multiplied and accumulated, and the obtained value is the local convolution result at the corresponding position. Since the traditional convolution needs to traverse the whole image, the computational complexity is high.

As shown in the lower part of Fig. 3, based on the Toeplitz matrix product convolution, each 3 × 3 process matrix obtained by the convolution kernel traversal matrix is expanded in line with row order to obtain a 4 × 1 × 9 row matrix, forming a large matrix *H*. Then, the convolution matrix is expanded into a 9 × 1 column vector *X* in line with row arrangement order. The product of the large matrix *H* constructed by the convolution kernel and the column vector *X* to be constructed by the convolution matrix effectively replaces the convolution computation. Specifically, the convolution kernel matrix *H* is composed of 6 small matrices, which are respectively the matrix in the red box and the matrix in the yellow box, as well as the zero matrix in the two white parts.

In Fig. 3, the matrix in the red box conforms to the definition form of the Toeplitz matrix. Similarly, the matrix in the yellow box and the zero matrix are Toeplitz matrices. Therefore, the convolution kernel matrix constructed is a large Toeplitz matrix composed of several small Toeplitz matrices. The product of the Toeplitz matrix is used to replace the traditional convolution operation. The convolution kernel is directly constructed into the convolution kernel matrix without traversing the image in order of step size, and the product of the two matrices is calculated to reduce the computational complexity.

Definition 2 Toeplitz matrix: A matrix with the same elements on each diagonal line from the top left to the bottom right is a Toeplitz matrix, which has the properties $A_{i,j} = A_{i + 1,j + 1} = a_{i - j}$. Mathematically,

9 $$ A = \left( {\begin{array}{*{20}c} {a_{0} } & {a_{ - 1} } & {a_{ - 2} } & {...} & {...} & {a_{{ - \left( {n - 1} \right)}} } \\ {a_{1} } & {a_{0} } & {a_{ - 1} } & \ddots & {} & \vdots \\ {a_{2} } & {a_{1} } & \ddots & \ddots & \ddots & \vdots \\ \vdots & \ddots & \ddots & \ddots & {a_{ - 1} } & {a_{ - 2} } \\ \vdots & {} & \ddots & {a_{1} } & {a_{0} } & {a_{ - 1} } \\ {a_{n - 1} } & {...} & {...} & {a_{2} } & {a_{1} } & {a_{0} } \\ \end{array} } \right) $$

#### Toeplitz convolution kernel matrix Construction

In order to replace the convolution calculation with the Toeplitz matrix multiplication operation, the convolution kernel matrix *H* is constructed as the Toeplitz convolution kernel matrix *H*_*t*_. Given any convolution kernel matrix as follow:

10 $$ H = \left( {\begin{array}{*{20}c} {h_{11} } & {h_{12} } & \cdots & {h_{1D} } \\ {h_{21} } & {h_{22} } & \cdots & {h_{2D} } \\ \vdots & \vdots & \vdots & \vdots \\ {h_{C1} } & {h_{C2} } & \cdots & {h_{CD} } \\ \end{array} } \right) $$

The corresponding construction steps of the Toeplitz convolution kernel matrix are as follows:

1. A small Toeplitz matrix is generated from each row element of the convolution kernel matrix. Since the size of the convolution kernel matrix is *C* × *D*, the convolution kernel matrix *H* is divided into *C* Toeplitz matrices: *H*_0_, *H*_1_, *H*_2_, *H*_3_, …, *H*_*c*-1_, where *H*_0_ is the zero interpolation of the element *h*_11_ in the first row and first column of *H*, the number of inserted zeros is the number of columns in the convolution kernel matrix *H* minus 1, and the interpolation result is taken as the first row of *H*_0_. Then *h*_12_ is interpolated as the second row according to the properties of the Toeplitz matrix until the 2 × *D*-1 rows are formed and the *H*_0_ construction is completed. By analogy, *H*_*i*_ is the matrix obtained by interpolating the $i - 1$ row elements of *H*. For example, the convolution kernel matrix is $$H = \left[ {\begin{array}{*{20}c} 1 & 2 \\ 3 & 4 \\ \end{array} } \right]$$, then *H* is divided into two matrices $$H_{0} = \left[ {\begin{array}{*{20}c} 1 & 0 \\ 2 & 1 \\ 0 & 2 \\ \end{array} } \right]$$ and $$H_{1} = \left[ {\begin{array}{*{20}c} 3 & 0 \\ 4 & 3 \\ 0 & 4 \\ \end{array} } \right]$$.
2. The small Toeplitz matrix obtained in Step (1) is formed into a large Toeplitz matrix:
  11 $$ H_{t} = \left( {\begin{array}{*{20}c} {H_{0} } & 0 & {...} & 0 & 0 \\ {H_{1} } & {H_{0} } & \ddots & \vdots & \vdots \\ {H_{2} } & {H_{1} } & \ddots & 0 & 0 \\ \vdots & {H_{2} } & \ddots & {H_{0} } & 0 \\ {H_{c - 2} } & \vdots & \ddots & {H_{1} } & {H_{0} } \\ {H_{c - 1} } & {H_{c - 2} } & \vdots & {H_{2} } & {H_{1} } \\ 0 & {H_{c - 1} } & {H_{c - 2} } & \vdots & {H_{2} } \\ 0 & 0 & {H_{c - 1} } & {H_{c - 2} } & \vdots \\ \vdots & \vdots & \vdots & {H_{c - 1} } & {H_{c - 2} } \\ 0 & 0 & 0 & \cdots & {H_{c - 1} } \\ \end{array} } \right) $$
  In the example in Step (1), $$H_{t} = \left[ {\begin{array}{*{20}c} {H_{0} } & 0 \\ {H_{1} } & {H_{0} } \\ 0 & {H_{1} } \\ \end{array} } \right]$$ is obtained by Eq. (11), where 0 represents a zero matrix of 3 × 2.

#### Toeplitz matrix convolution

After obtaining the Toeplitz convolution kernel matrix from “Toeplitz convolution kernel matrix construction” section 8, the traditional convolution can be replaced by the Toeplitz matrix multiplication using Eq. (12).

12 $$ X*H = H_{t} \times X_{T} $$

where $$X = \left( {\begin{array}{*{20}c} {x_{11} } & {x_{12} } & \cdots & {x_{1B} } \\ {x_{21} } & {x_{22} } & \cdots & {x_{2B} } \\ \vdots & \vdots & \vdots & \vdots \\ {x_{A1} } & {x_{A2} } & \cdots & {x_{AB} } \\ \end{array} } \right)$$ denotes the matrix to be convolved, $$H = \left( {\begin{array}{*{20}c} {h_{11} } & {h_{12} } & \cdots & {h_{1D} } \\ {h_{21} } & {h_{22} } & \cdots & {h_{2D} } \\ \vdots & \vdots & \vdots & \vdots \\ {h_{C1} } & {h_{C2} } & \cdots & {h_{CD} } \\ \end{array} } \right)$$ denotes the convolution kernel, *H*_*t*_ is the Toeplitz convolution kernel matrix in "Toeplitz convolution kernel matrix construction" section, and *X*_*T*_ is the column vector obtained by arranging all the elements of *X* in row order. Using the full convolution method, the matrix to be convolved is filled with zeros, and the result returns all the data after convolution. The row number of the convolution result matrix is *M* = *A* + *C − *1 and the column number of the convolution result matrix is *N* = *B* + *D − *1.

For example, when $$X = \left[ {\begin{array}{*{20}c} 5 & 6 \\ 7 & 8 \\ \end{array} } \right]$$, then $$X_{T} = \left[ {\begin{array}{*{20}c} 5 & 6 & 7 & 8 \\ \end{array} } \right]^{T}$$, and the results that use convolution calculation is $$X*H = \left[ {\begin{array}{*{20}c} 5 & 6 \\ 7 & 8 \\ \end{array} } \right]*\left[ {\begin{array}{*{20}c} 1 & 2 \\ 3 & 4 \\ \end{array} } \right] = \left[ {\begin{array}{*{20}c} 5 & {16} & {12} \\ {22} & {60} & {40} \\ {21} & {52} & {32} \\ \end{array} } \right]$$. The result that uses convolution operation based on the Toeplitz matrix is.

$$H_{t} \times X_{T} = \left[ {\begin{array}{*{20}c} {H_{0} } & 0 \\ {H_{1} } & {H_{0} } \\ 0 & {H_{1} } \\ \end{array} } \right] \times \left[ {\begin{array}{*{20}c} 5 & 6 & 7 & 8 \\ \end{array} } \right]^{T} = \left[ {\begin{array}{*{20}c} 5 & {16} & {12} & {22} & {60} & {40} & {21} & {52} & {32} \\ \end{array} } \right]^{T}$$.

Then the calculated column vector is rewritten into a 3 × 3 matrix according to *M* = *A* + *C − *1 = 3 and *N* = *B* + *D − *1 = 3, which is the same as the results of the convolution calculation.

We use the Toeplitz matrix multiplication to effectively replace the convolution operation. In terms of time complexity, the input time-domain image size is *A* × *B*, and the convolution kernel size is *C* × *D*. The convolution operation requires the convolution kernel to continuously traverse the time domain image and calculate *A* × *B* × *C* × *D* times multiplication.

When using the Toeplitz matrix multiplication, it only needs to calculate the matrix multiplication once. It is realized from Fig. 3 that there are a large number of zeros in each row of the matrix which does not need to be calculated. Thus, the actual calculation of each row is *C* × *D*, the row number is the time of convolution kernel traverses, and approximately multiply *A* × *B* × *C* × *D* times. Therefore, during a calculation, the calculation amount of the two methods is roughly the same. However, when a new time-domain image is inputted into the traditional convolution each time, there are a large number of shift operations in the calculation, which greatly increases the calculation time.

Although it takes some time to construct the Toeplitz matrix, Toeplitz matrix multiplication only needs to construct the corresponding Toeplitz matrix once according to the given convolution kernel, and then can directly perform the matrix multiply calculation on all the input time-domain images to obtain the convolution result. In this way, for the datasets with a large number of sample sets and test sets, the convolution operation time will be greatly reduced.

### T-CNN model classification

When a CNN model is used for classification, its fully connected layers perform convergence operations, and a given loss function is required. In this paper, the Triplet network is introduced into the loss function, and then the T-CNN model is proposed.

Let the sample set of *m* samples is $\left\{ {\left( {x^{\left( 1 \right)} ,y^{\left( 1 \right)} } \right),\left( {x^{\left( 2 \right)} ,y^{\left( 2 \right)} } \right),...,\left( {x^{\left( m \right)} ,y^{\left( m \right)} } \right)} \right\}$, there are *n* classes in these samples, which $y^{\left( i \right)}$ represents the expected output of $x^{\left( i \right)}$, and the loss function of CNNs is shown as Eq. (13):

13 $$ R\left( {\omega ,b} \right) = \frac{1}{m}\sum\limits_{{i = 1}}^{m} {\left( {\frac{1}{2}\left\| {p_{{\omega ,b}} \left( {x^{{\left( i \right)}}  - y^{{\left( i \right)}} } \right)} \right\|^{2} } \right)}  $$

where $\omega$ is the weight of each neuron, $b$ is the bias, and $p_{\omega ,b} \left( {x^{i} } \right)$ is the actual output of the sample. The CNN model continuously adjusts the parameter $\omega$ and $b$ by training to minimize $R\left( {\omega ,b} \right)$. Equation (13) is the square loss function of the traditional convolutional neural network model, which only considers the category of the image itself and does not consider the differences between different categories. Therefore, we will improve it later.

The CNN uses the gradient descent method to adjust the parameter $R(\omega ,b)$, as shown in Eqs. (14) and (15):

14 $$ \omega_{ij} = \omega_{ij} - a\frac{\partial }{{\partial \omega_{ij} }}R\left( {\omega ,b} \right) $$

15 $$ b_{ij} = b_{ij} - a\frac{\partial }{{\partial b_{ij} }}R\left( {\omega ,b} \right) $$

where *a* is the learning rate and $R\left( {\omega ,b} \right)$ is the CNN loss function. Equations (14) and (15) are used to update the values of network parameters $\omega$ and $b$. The calculation method is the gradient descent method. In other words, the value of $\omega$ and $b$ can be obtained when the derivative of the loss function is 0.

**Figure 4:**
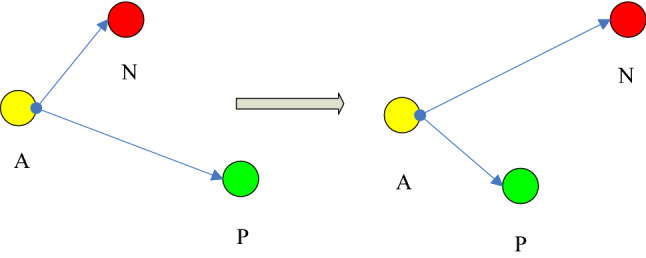
Adjustment chart of feature difference. (It is used to illustrate the triple Loss function proposed in this paper. The software is Microsoft Office Visio).

In order to improve the classification accuracy, the Triplet network is introduced into the CNN loss function for constraint, and a T-CNN model based on the Triplet loss function is proposed. The idea of the T-CNN model is to input three-time domain images at a time, two of which belong to the same class and one belongs to another class. The T-CNN model can obtain the feature of the time domain images by training and can obtain the feature difference function $$L_{1}$$ of two-time domain images from the same class and the feature difference function $$L_{2} $$ of two-time domain images from different classes. Then $$L_{1} $$ and $$L_{2} $$ are used to adjust the parameters of the T-CNN model. $$L_{1} $$ and $$L_{2}$$ are shown in Eqs. (16 )and (17) respectively:

16 $$  L_{1}  = \frac{1}{2}\left\| {p_{{\omega ,b}}^{{\left( {l_{1} } \right)}}  - p_{{\omega ,b}}^{{\left( {l_{2} } \right)}} } \right\|^{2}  $$

17 $$  L_{2}  = \frac{1}{2}\min \left\| {n_{{\omega ,b}}^{{\left( l \right)}}  - p_{{\omega ,b}}^{{\left( {l_{i} } \right)}} } \right\|^{2} ,\left( {i = 1,2} \right)  $$

where $p_{\omega ,b}^{{\left( {l_{i} } \right)}}$ is the output value of the same class and $n_{\omega ,b}^{\left( l \right)}$ is the output value of the different classes. The image feature difference functions are shown in the adjustment Eq. (18).

18 $$ L_{T} = \max (0,L_{1} - L_{2} + \gamma ) $$

where $\gamma$ represents the minimum distance of the difference function between different classes and between classes (set to 0.1 in this paper). In the experiment of this paper, the comparison experiment was conducted by changing the value of $\gamma$, and the value of $\gamma$ was 0.01, 0.05, 0.1, 0.2 and 0.5 respectively. The experiment found that 0.1 was the best experimental result. In each reverse iteration, *L*_*T*_ gradually approaches zero. As shown in Fig. 4, when the feature difference function *L*_1_ of the same class of images is greater than the feature difference function *L*_2_ of different classes of images minus the parameter *α*, *L*_*T*_ is greater than zero, and the model is adjusted in reverse to make *L*_1_ smaller and *L*_2_ larger. Reference ^21^ has verified that the Triplet loss function can make samples of the same kind close to each other and samples of different kinds far from each other.

In Fig. 4, *A* and *P* belong to the same class, while *N* does not belong to the same class as *A* and *P*. Before the adjustment, the distance between *A* and *P* is greater than that between *A* and *N*, and the difference function *L*_*T*_ is greater than zero. Thus, the model parameters need to be adjusted in reverse. After the adjustment, the distance between *A* and *N* becomes larger, while the distance between *A* and *P* becomes smaller.

According to Eqs. (16) and (17), in each reverse iteration, it can be seen that *L*_*1*_ will make the feature difference of the same class smaller, while *L*_*2*_ will make the feature difference of different classes larger. On this basis, a Triplet loss function is proposed as shown in Eq. (19):

19 $$ L\left( {\omega ,b} \right) = R\left( {\omega ,b} \right) + \alpha L_{1} - \beta L_{2} $$

where $R\left( {\omega ,b} \right)$ denotes the CNN square loss function, $\alpha$ and $\beta$ are the weight proportion coefficients greater than zero. In the experiment, we tested the values of $\alpha$ and $\beta$. The values of $\alpha$ were 0.1, 0.01, 0.3, 0.4 and so on, and the values of $\beta$ were 0.9, 0.99, 0.7, 0.6 and so on. After several experiments, it was found that the values of $\alpha$ and $\beta$ were 0.4, 0.6 respectively, and the experimental effect was the best. *L*_*1*_ is the feature difference function of the same class, and *L*_*2*_ is the feature difference function of different classes. Therefore, the new residual error of each layer by the backpropagation algorithm is as follows:

20 $$ \omega_{ij} = \omega_{ij} - a\frac{\partial }{{\partial \omega_{ij} }}L\left( {\omega ,b} \right) $$

21 $$ b_{ij} = b_{ij} - a\frac{\partial }{{\partial b_{ij} }}L\left( {\omega ,b} \right) $$

The T-CNN model based on the Triplet network adds the feature difference function between the same class and the feature difference function between different classes into a cross-entropy loss function, which is conducive to allow the parameters to extract features with larger differences more quickly in the process of parameter weight adjustment. The partial derivative of $L\left( {\omega ,b} \right)$ can make the backpropagation residual calculation to obtain new parameters $\omega$ and $b$. Each iteration is more inclined to the direction of gradient descent, which can make the model converge faster and improve the classification efficiency.

**Figure 5:**
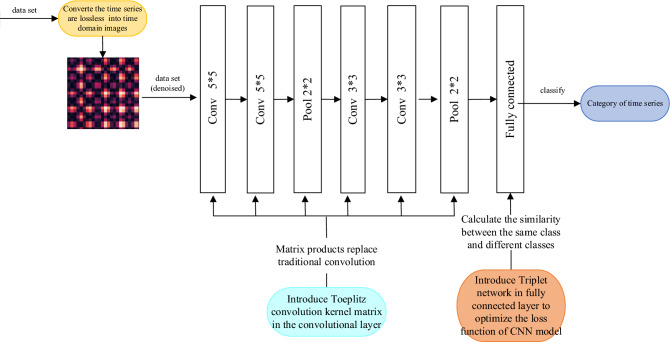
The structure of the T-CNN classification model.


The T-CNN model structure used in this paper is 5 × 5 convolution of 128 neurons in the first layer, 5 × 5 convolution of 128 neurons in the second layer, maximum pooling layer of 2 × 2 in the third layer, 3 × 3 convolution of 256 neurons in the fourth layer, 3 × 3 convolution of 256 neurons in the fifth layer, maximum pooling layer of 2 × 2 in the sixth layer, 1024 neurons in the full connection layer in the seventh layer. The loss function is the Triplet-based loss function, the activation function is the sigmoid function, and the value range of the function is (0,1). Figure 5 is the model structure. T-CNN time series classification algorithm is shown in Algorithm 1.

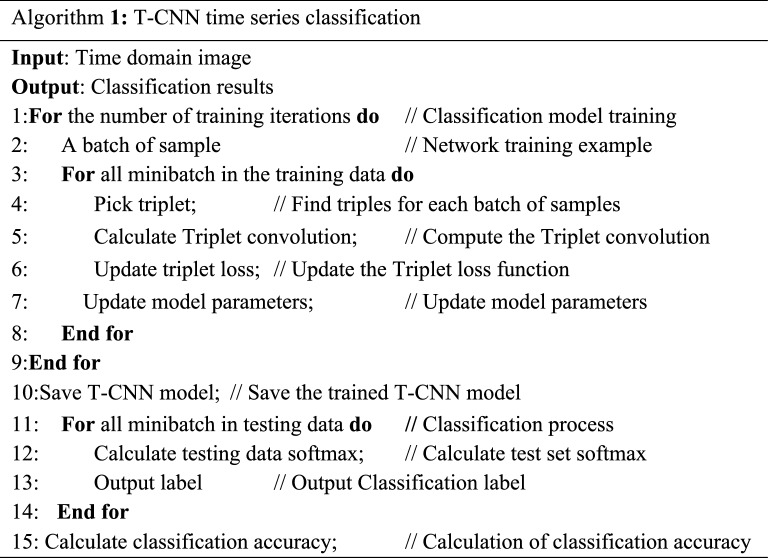


## Experiments

The experimental data set adopts 6 data sets of UCR2018 and microquake data sets, which contains three types of time series event waveforms of mine microseismic signals. The dataset includes time series data of different sizes, lengths and categories. The size of the training set accounts for 60% of the total data set, and the size of the test set accounts for 40% of the total data set, and the model training framework is Tensorflow. The hardware and software environment of the experiment are shown in Table 1.

**Table 1:** Software and hardware environment.

CPU	Intel Core(TM)i7-7500U
Memory	8 GB
Hard disk	1 TB
OS	Windows 8.1 (64bit)
Programming language	Python

The experiment data sets are shown in Table 2. After applying the wavelet denoising method in this paper, the type and length of the microquake dataset containing noise will not change, but the length itself is unequal, so it will not affect the structure of the dataset.

**Table 2:** The experiment data sets.

Data sets	Train	Test	Class	Length	Noises
BME	72	180	3	128	Denoised
ItalyPowerDemand	438	1096	2	24	Denoised
SyntheticControl	240	600	6	60	Denoised
Cricket_X	312	780	12	300	Denoised
SwedishLeaf	450	1125	15	128	Denoised
WordSynonyms	362	905	25	270	Denoised
Microquake	4000	10,000	3	Unequal	Noised

In order to prevent local over convergence, the convolutional neural network structure is equipped with two convolutional layers. The convolutional layer 1 uses a convolution kernel of 5 × 5 size, and the maximum pooling window size is 3 × 3. The convolutional layer 2 uses a convolution kernel of 3 × 3 size, and the maximum pooling window size is 2 × 2. The convolutional layer 2 is followed by a standard fully connected layer. The activation function of this layer uses ReLU which makes the CNN model converge quickly. The loss function uses the Triplet loss function. The time-domain image output by the T-CNN model is *p* = {*p*_1_, *p*_2_, *p*_3_}, where *p*_1_, *p*_2_, and *p*_3_ represent the probabilities of three classes in the data set respectively. *class* = *T*(*max*(*p*)) is used for judgment classes, where the function T is a threshold function, and the threshold is set to 0.8 after repeated experiments. When *max*(*p*) is greater than the threshold set by the function *T*, the class of time-domain image is output. In order to make the experimental results more reliable, the experiment uses a tenfold cross validation, and the final result is an average of 10 times. The T-CNN model adjusts itself to the optimal state through continuous forward conduction, in which the main adjustable parameters are the learning rate and the iteration times. During this experiment, the learning rate is set to 0.005, and the iteration time is adjusted.

### Comparison of classification accuracy

**Figure 6:**
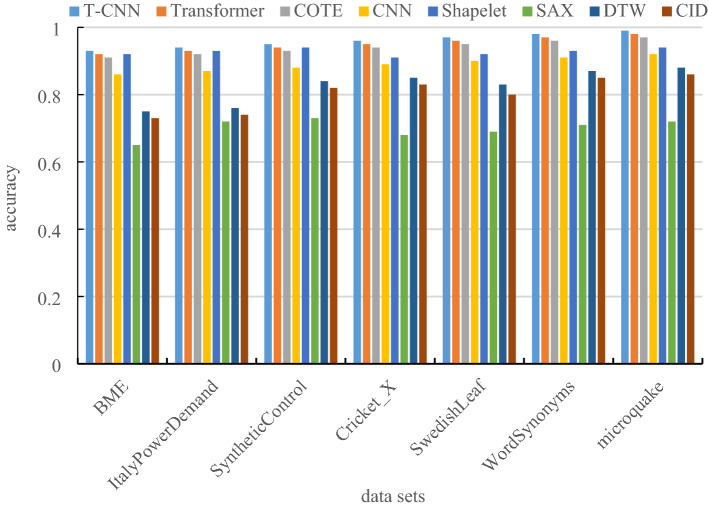
Comparison of classification accuracy.

The accuracy is the proportion of the number of samples correctly classified to the total number of samples. Figure 6 is the comparison of classification accuracy by eight different classification models. The compared methods are Transformer, symbolic aggregation approximation (SAX), Shapelet, Dynamic Time Warping (DTW), Collective of Transformation-based Ensembles (COTE), Complexity Invariant distance (CID) and CNN classification method.

The experimental comparison results of classification accuracy under different data sets are shown in Table 3.

**Table 3:** Comparison of classification accuracy table.

	T-CNN	Transformer	COTE	CNN	Shapelet	SAX	DTW	CID
BME	0.93	0.92	0.91	0.86	0.92	0.65	0.75	0.73
ItalyPowerDemand	0.94	0.93	0.92	0.87	0.93	0.72	0.76	0.74
SyntheticControl	0.95	0.94	0.93	0.88	0.94	0.73	0.84	0.82
Cricket_X	0.96	0.95	0.94	0.89	0.91	0.68	0.85	0.83
SwedishLeaf	0.97	0.96	0.95	0.90	0.92	0.69	0.83	0.80
WordSynonyms	0.98	0.97	0.96	0.91	0.93	0.71	0.87	0.85
Microquake	0.99	0.98	0.97	0.92	0.94	0.72	0.88	0.86

From Fig. 6 and Table 3, it is known that the T-CNN model converts the time series into the time domain images by using the Gram matrix, it can completely retain the time feature of the time series, and its classification accuracy is significantly better than other methods.

### Comparison of classification precision

**Figure 7:**
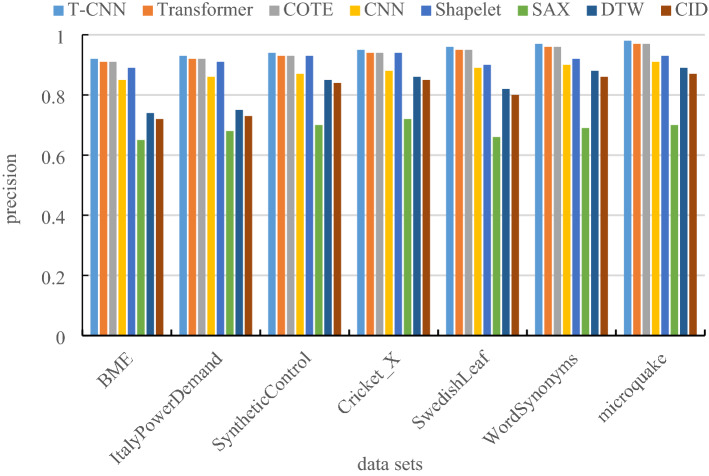
Comparison of classification precision.

The precision is the proportion of samples predicted to be one class that is truly in that class, which is given by:

22 $$ P_{i} = \frac{{n_{ii} }}{{\mathop \sum \nolimits_{j = 1}^{{n_{c} }} n_{ji} }} $$

where $n_{ij}$ is the number of samples predicted as the *j-*th class in class *i*, and $n_{c}$ is the number of sample classes. Then calculate the average precision of all classes to get the average precision of the classification. Figure 7 is the comparison of classification precision.

The experimental comparison results of classification precision under different data sets are shown in Table 4.

**Table 4:** Comparison of classification precision table.

	T-CNN	Transformer	COTE	CNN	Shapelet	SAX	DTW	CID
BME	0.92	0.91	0.90	0.85	0.89	0.65	0.74	0.72
ItalyPowerDemand	0.93	0.92	0.91	0.86	0.91	0.68	0.75	0.73
SyntheticControl	0.94	0.93	0.92	0.87	0.93	0.7	0.85	0.84
Cricket_X	0.95	0.94	0.93	0.88	0.94	0.72	0.86	0.85
SwedishLeaf	0.96	0.95	0.94	0.89	0.9	0.66	0.82	0.8
WordSynonyms	0.97	0.96	0.95	0.9	0.92	0.69	0.88	0.86
Microquake	0.98	0.97	0.96	0.91	0.93	0.7	0.89	0.87

From Fig. 7 and Table 4, due to the improved loss function of the T-CNN model, the classification precision is significantly better than other methods.

### Comparison of classification recall

**Figure 8:**
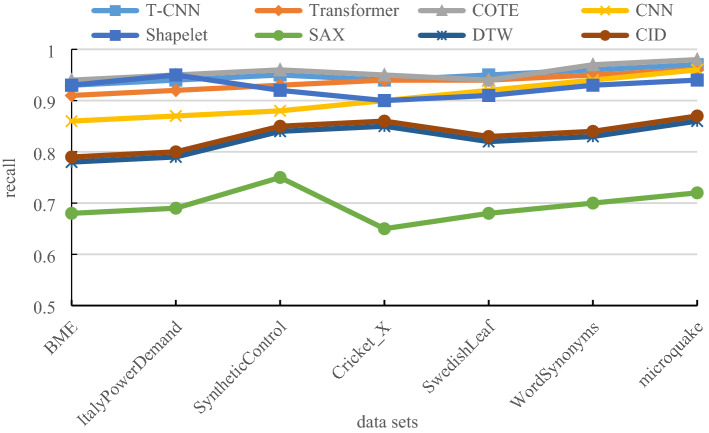
Comparison of classification recall.

The recall is the proportion of samples detected in one class, which is given by:

23 $$ R_{i} = \frac{{n_{ij} }}{{\mathop \sum \nolimits_{j = 1}^{{n_{c} }} n_{ij} }} $$

where $n_{ij}$ is the number of samples predicted as the *j-*th class in class *i*, and $n_{c}$ is the number of sample classes. Then calculate the average recall of all classes to get the average recall of the classification. Figure 8 is the comparison of classification recall.

The experimental comparison results of classification recall under different data sets are shown in Table 5.

**Table 5:** Comparison of classification recall.

	T-CNN	Transformer	COTE	CNN	Shapelet	SAX	DTW	CID
BME	0.93	0.91	0.94	0.86	0.93	0.68	0.78	0.79
ItalyPowerDemand	0.94	0.92	0.95	0.87	0.95	0.69	0.79	0.8
SyntheticControl	0.95	0.93	0.96	0.88	0.92	0.75	0.84	0.85
Cricket_X	0.94	0.94	0.95	0.9	0.9	0.65	0.85	0.86
SwedishLeaf	0.95	0.94	0.94	0.92	0.91	0.68	0.82	0.83
WordSynonyms	0.96	0.95	0.97	0.94	0.93	0.7	0.83	0.84
Microquake	0.97	0.96	0.98	0.96	0.94	0.72	0.86	0.87

From Fig. 8 and Table 5, the T-CNN model uses a Gram matrix to convert the time series without loss and improves the loss function, therefore, the classification recall is close to that of the COTE method and superior to other methods.

### Comparison of accuracy between Toeplitz convolution and traditional convolution

**Figure 9:**
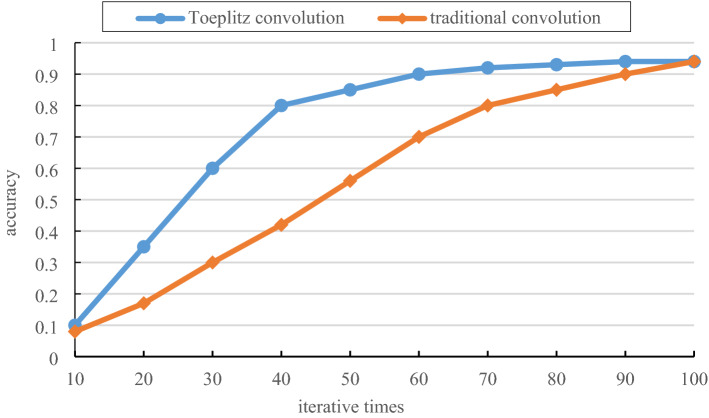
Comparison of convolution running time.

In this section, the accuracy of convolution based on the Toeplitz matrix product and traditional convolution is compared. In the experimental process, the size convolution kernel is taken to calculate the convolution, and the time series length is successively intercepted from 10 to 100. The time domain image size transformed based on the Gram matrix is from 10*10 to 100*100. A comparison of the accuracy of Toeplitz convolution and traditional convolution for convolution of time-domain images of different sizes is shown in Fig. 9. The abscissa is the number of iterations, and the ordinate is the accuracy.

From Fig. 9, after several experiments, after 70 iterations, the training set can achieve a good effect, and the accuracy on the test set reaches 90% and tends to be stable. the accuracy of both traditional convolution and Toeplitz convolution changes gently between the image size of 10 and 40 in the time domain. When the image size of more than 40 in the time domain, the accuracy of the two convolutions increases faster. On the whole, when the image size is between 10*10 and 100*100, the accuracy of Toeplitz convolution is higher than that of traditional convolution operation, so the convergence of Toeplitz convolution is faster. Therefore, the Toeplitz convolution proposed in this paper can speed up the training of the model.

## Conclusion

This paper proposes a T-CNN time series classification method based on the Gram matrix. The method firstly denoises the time series by using a wavelet threshold, and then converts the loss-free time series into time images by the Gram matrix, time attribute is retained to improve classification accuracy. And on this basis, the T-CNN model is proposed to introduce the Toeplitz convolutional kernel matrix into the convolution layer, replaces the convolutional operation with the multiplication of two matrices, and introduces the feature difference function of the same class and different classes to improve the CNN loss function of the fully connected layer, and the classification efficiency is improved. Experimental results show that the proposed T-CNN time series classification method is suitable for processing time series classification, and the classification accuracy, precision and recall are better than the existing methods.
